# A Safe Technique to Remove the Hip Spica

**DOI:** 10.5704/MOJ.2003.020

**Published:** 2020-03

**Authors:** A Mazelan, AWC Lam, MZA Albaker

**Affiliations:** Department of Orthopaedic Surgery, University of Malaya, Kuala Lumpur, Malaysia

Dear Chief Editor,

Hip spica is commonly used in the management of developmental dysplasia of the hip^1^ and femur fractures in young children^2^. Removal of the spica cast is commonly performed in the outpatient clinic or under general anaesthesia. The procedure is not free from complications. Physical or thermal injury to the skin due to the moving blade of the oscillating saw blade may occur especially around the groin area where it is difficult to estimate the thickness of the cast material^3^. We would like to share a method of hip spica removal that we feel is associated with lower risk of skin injury.

**Fig. 1:**
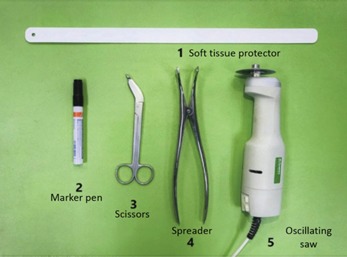
Tools required for bivalving hip spica.

**Fig. 2:**
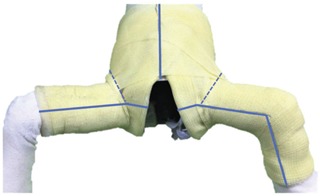
Anterior aspect of hip spica. The 3 solid blue lines depicting the 3 cuts by oscillating saw which avoid groin creases (dotted lines).

First, we should prepare the basic instruments for hip spica cast removal (Fig. 1). The child with spica cast is then placed in supine position. Three lines of cut are drawn over the anterior surface of the spica cast (Fig. 2). The first line is drawn from the midpoint of the superior rim vertically downwards to the groin opening. The shorter limb of the spica cast is usually on the contra-lateral to the unstable hip or fractured femur. The second line is drawn from the midpoint of the inner distal rim, extending horizontally to the groin opening. The longer limb of the cast is usually on the ipsilateral side of the pathological condition. A third anterior line is drawn from the midpoint of the inner ankle, extending proximally crossing the medial aspect of knee, and horizontally towards the groin opening. Cutting the spica cast along these lines would circumvent the groin crease, which is one of the common sites of skin injury. In addition, soft tissue protector can be inserted between the cast and skin along these lines to further reduce the risk of skin injury. Once the cuts had been performed, a spreader is used to ensure that the cuts were complete (full thickness).

**Fig. 3:**
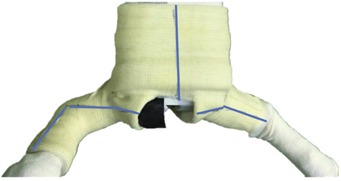
Posterior aspect of hip spica. The 3 solid blue lines depicting the 3 cuts by oscillating saw.

The child with the cast is then put in prone position. Three more lines are drawn on the posterior surface of the cast (Fig. 3). The first line is a vertical line extending from the midpoint of the superior rim, caudally towards the groin opening. The second line on the shorter limb is a horizontal line extending from the midpoint of the distal rim to the groin opening. The third line extends from the midpoint of the distal rim of the ankle, proximally on the lateral aspect of the knee, extending horizontally to the groin opening. Once these cuts have been completed, the cast can be removed.

In conclusion, the advantage of this technique is that it avoids cuts over the groin crease area. Thickness of cast material over this region is generally not uniform, and the area may be very close to the wound and metal wires used to fix pelvic osteotomies. Moreover, most of the cuttings are approaching the trunk and limbs from the front. Soft tissue tends to fall back due to gravity, and this would further reduce the risk of skin injury.
